# Genetic polymorphisms in inflammatory response genes and their associations with breast cancer risk

**DOI:** 10.3325/cmj.2014.55.638

**Published:** 2014-12

**Authors:** Zhi Wang, Qiu-Lian Liu, Wu Sun, Chun-Jing Yang, Lei Tang, Xian Zhang, Xiao-Ming Zhong

**Affiliations:** 1 Department of Oncology, Jiujiang First People’s Hospital, Jiujiang, Jiangxi, China; 2 Department of Laboratory, Jiujiang First People’s Hospital, Jiujiang, Jiangxi, China; 3 Department of Radiotherapy, Jiangxi Province Cancer Hospital, Nanchang, Jiangxi, China

## Abstract

**Aim:**

To explore the association of *NFKB1* c.-798_-795delATTG (rs28362491), *NFKBIA* c.-949C>T (rs2233406), *IL-8* c.-352A>T (rs4073), *IL-10* c.-854T>C (rs1800871), *TNF* c.-418G>A (rs361525), and *TNF* c.-488G>A (rs1800629) polymorphisms with breast cancer risk in an East Chinese population.

**Methods:**

We conducted a case-control study including 975 study participants (474 breast cancer patients and 501 female controls without cancer) and genotyped the polymorphisms employing polymerase chain reaction-restriction fragment length polymorphism (PCR-RFLP). Logistic regression was used to assess the association of the polymorphisms with breast cancer risk.

**Results:**

We found that the ins/del and del/del genotypes of *NFKB1* polymorphism and TT genotype of *IL-10* polymorphism significantly increased breast cancer risk (*NFKB1* ins/del odds ratio [OR] 1.69, 95% [CI] 1.23-2.33, *P* = 0.001; *NFKB1* del/del OR 2.42, 95% CI 1.72-3.42, *P* < 0.001; *IL-10* TT OR 2.36, 95% CI 1.58-3.52, *P* < 0.001). On the other hand, the TT genotype of *IL-8* polymorphism, GA and AA genotypes of *TNF* c.-418G>A polymorphism, and GA genotype of *TNF* c.-488G>A polymorphism significantly reduced breast cancer risk (*IL-8* TT OR 0.48, 95% CI 0.33-0.72, *P* < 0.001; *TNF* c.-418 GA OR 0.58, 95% CI 0.41-0.80, *P* = 0.001; *TNF* c.-418 AA OR 0.38, 95% CI 0.14-0.98, *P* = 0.044; *TNF* c.-488 GA OR 0.68, 95% CI 0.48-0.96, *P* = 0.029). When stratified by menopausal status, the CT genotype of *NFKBIA* polymorphism significantly reduced the risk among pre-menopausal women (OR 0.63, 95% CI 0.40-0.99, *P* = ,043), but not among post-menopausal women.

**Conclusions:**

*NFKB1*, *NFKBIA*, *IL-8*, *IL-10*, and *TNF* polymorphisms could serve as useful predictive biomarkers for breast cancer risk among women in East China.

Breast cancer is the most frequent form of cancer and leading cause of cancer-related deaths among women around the world (1). The cancer accounts for almost one quarter of new cancer cases annually (2), and the incidence continues to increase rapidly, both in China and worldwide (3). Although it has been well-established that breast carcinogenesis is a result of the complex interactions between multiple environmental and genetic factors, the mechanisms of the oncogenesis at the molecular level remain poorly understood. Genetic factors can serve as a susceptibility variable for breast cancer development, and their identification can help to reduce the incidence of breast cancer (4). However, several breast cancer susceptibility genes identified so far, such as *BRCA1* and *BRCA2*, account for only less than 5% of the total breast cancer incidence (5).

Single nucleotide polymorphisms (SNPs) have been extensively investigated for their associations with the risk of various cancers (6-11). As inflammation is caused by a molecular network underlying breast carcinogenesis (12), we propose that SNPs within inflammatory response genes could modify breast cancer predisposition risk. The associations of various inflammatory response gene polymorphisms with breast cancer risk in the Chinese population, especially the East Chinese population, have been understudied. In the current study, we investigated the associations of *NFKB1* c.-798_-795delATTG (rs28362491), *NFKBIA* c.-949C>T (rs2233406), *IL-8* c.-352A>T (rs4073), *IL-10* c.-854T>C (rs1800871), *TNF* c.-418G>A (rs361525), and *TNF* c.-488G>A (rs1800629) polymorphisms with breast cancer risk in East China. Since all these polymorphisms are located in the promoter region, they could affect the transcriptional activity of the gene, resulting in enhanced or reduced cDNA, and eventually protein levels, among their carriers (6,7,13). In addition, despite the relatively well established associations of the polymorphisms with cancer risks in other populations (6-9), little is known about their association with breast cancer risk in East China population, which further motivated us to undertake this research.

## Patients and methods

### Study participants and ethical considerations

A total of 1032 female study participants – 514 breast cancer patients and 518 controls without cancer were identified at the Jiujiang First People’s Hospital. 474 breast cancer patients and 501 female controls without cancer agreed to participate in the study. The participants were interviewed by trained professionals and data related to smoking, oral contraceptive use, and menopausal status were collected. The patients’ histopathological types and cancer grading were retrieved from their medical records. All the participants were Han Chinese. The study received approval from the Ethics of Human Research Board of Jiujiang First People’s Hospital. Informed consent was obtained from the participants before inclusion in the study.

### Genotyping

Polymorphisms were genotyped on the DNA isolated from the peripheral blood samples using polymerase chain reaction-restriction fragment length polymorphism (PCR-RFLP) technique and the genotypes were verified by direct sequencing of PCR products. For *NFKB1* c.-798_-795delATTG (rs28362491), the PCR primers used were 5′-TGG GCA CAA GTC GTT TAT GA-3′ and 5′-CTG GAG CCG GTA GGG AAG-’3 (6) and the annealing temperature was 63.5°C. The PCR product 281 bp (deletion allele) or 285 bp (insertion allele) was digested with PflMI (Van91I) restriction enzyme. The insertion genotype was identified as 2 bands on agarose gel, at 240 bp and 45 bp.

For *NFKBIA* c.-949C>T (rs2233406) polymorphism, the forward primer was 5′-GGT CCT TAA GGT CCA ATC G-3′ and the reverse primer was 5′-GTT GTG GAT ACC TTG CAC TA-3′ (7). The annealing temperature was also 63.5°C; the 200 bp product was digested with BfaI restriction enzyme; and the CC genotype was identified as 180 + 20 bp bands.

For *IL-8* c.-352A>T (rs4073) polymorphism, the forward primer was 5′-CCA TCA TGA TAG CAT CTG T-3′ and the reverse primer was 5′-CCA CAA TTT GGT GAA TTA TTA A-3′ (8). The annealing temperature was 57°C; the 173 bp PCR product was digested with AseI restriction enzyme; and the AA genotype was identified as 152 + 21 bp bands.

For *IL-10* c.-854T>C (rs1800871) polymorphism, the forward primer was 5′-TGA GCA AAC TGA GGC ACA GAA AT-3′ and the reverse primer was 5′-GAC AAC ACT ACT AAG GCT CCT TTG GGA-3′ (14). The annealing temperature was 59°C; the 315 bp PCR product was digested with SspI restriction enzyme; and the TT genotype was identified as 291 + 24 bp bands.

For *TNF* c.-418G>A (rs361525) polymorphism, the primers used were 5′-AAA CAG ACC ACA GAC CTG GTC-3′ and 5′-CTC ACA CTC CCC ATC CTC CCG GAT C-3′ (15). Annealing temperature was 59°C; the 150 bp PCR product was digested with *Bam*HI restriction enzyme; and the GG genotype was identified as 130 + 20 bp bands.

For *TNF* c.-488G>A (rs1800629) polymorphism, the primers used were 5′-GAG GCA ATA GGT TTT GAG GGC CAT-3′ and 5′-GGG ACA CAC AAG CAT CAA G-3′ (15). The annealing temperature was 61°C; the 107 bp product was digested with NcoI restriction enzyme; and the GG genotype was identified as 87 + 20 bp bands.

### Statistical analysis

Statistical analysis was done by using SPSS, version 17.0 (SPSS Inc., Chicago, IL, USA) The differences in age, smoking habit, oral contraceptive use, menopausal status, and genotypic distribution between cases and controls were assessed using a χ^2^ test. Risk association between the polymorphisms and breast cancer was evaluated using logistic regression analysis. *P* values of <0.05 were considered significant.

## Results

There were no significant differences in mean age, smoking, oral contraceptives use, and menopausal status between patients and controls (Table 1).

**Table 1:** Demographic characteristics of cases with breast cancer and control participants

Variable	Cases	Controls	*P*
Mean age, mean ± standard deviation	59.1 ± 7.9	59.4 ± 8.0	0.567
Smoking, n			
Yes	138	137	0.540
No	336	364	
Oral contraceptive, n			
Use	135	159	0.268
No	339	342	
Menopausal status, n			
Pre	179	213	0.130
Post	295	288	
Histopathological type, n *			
IDC	346	-	-
DCIS	71	-	
ILC	57	-	
Grade, n^†^			
1	42	-	-
2	228	-	
3	204	-	

*IDC – invasive ductal carcinoma; DCIS – ductal carcinoma in situ; ILC – invasive lobular carcinoma.

^†^Grade 1 – well differentiated; Grade 2 – moderately differentiated; Grade 3 – poorly differentiated.

### Genotype distribution

Significant differences between cases and controls were observed for *NFKB1* ins/del and del/del genotypes, *IL-8* TT genotype, *IL-10* CC and TT genotypes, and *TNF* c.-418 and c.-488 GG and GA genotypes (Table 2). The two *TNF* polymorphisms were in strong linkage disequilibrium (R^2^ = 0.819). All the genotypic distributions followed Hardy-Weinberg equilibrium.

**Table 2:** Genotype distribution of the polymorphisms in cases with breast cancer and control participants

Gene	Genotype	Case, n/%	Controls, n/%	*P*
*NFKB1*	ins/ins	93/19.6	162/32.2	<0.001
	ins/del	210/44.3	216/43.1	0.708
	del/del	171/36.1	123/24.6	<0.001
*NFKBIA*	CC	288/60.8	297/59.3	0.637
	CT	147/31.0	162/32.3	0.657
	TT	39/8.2	42/8.4	0.930
*IL-8*	AA	192/40.5	186/37.1	0.281
	AT	231/48.7	213/42.5	0.052
	TT	51/10.8	102/20.4	<0.001
*IL-10*	CC	186/39.2	234/46.7	0.018
	CT	198/41.8	219/43.7	0.054
	TT	90/19.0	48/9.6	<0.001
*TNF* c.-418	GG	399/84.2	374/74.7	0.774
	GA	69/14.6	112/22.4	0.002
	AA	6/1.3	15/3.0	0.071
*TNF* c.-488	GG	404/85.2	397/79.2	0.015
	GA	66/13.9	95/19.0	0.034
	AA	4/0.8	9/1.8	0.206

### Association between the polymorphisms and breast cancer risk

Significant associations were observed for at least one genotype of all the polymorphisms, with the exception of *NFKBIA* polymorphism. *NFKB1* c.-798_-795delATTG ins/del and del/del genotypes, and *IL-10* c.-854 TT genotype were associated with increased breast cancer risk, while *IL8* c.-352 TT genotype, *TNF* c.-418 GA and AA genotypes, and c.-488 GA genotype were significantly associated with a reduced risk (Table 3).

**Table 3:** Association between the polymorphisms and breast cancer risk in cases with breast cancer and control participants

Gene		Genotype	Cases, n/%	Controls, n/%	Odds ratio (95% confidence interval)	*P*
*NFKB1*		ins/ins	93/19.6	162/32.2	-	-
		ins/del	210/44.3	216/43.1	1.69 (1.23-2.33)	0.001
		del/del	171/36.1	123/24.6	2.42 (1.72-3.42)	<0.001
*NFKBIA*		CC	288/60.8	297/59.3	-	-
		CT	147/31.0	162/32.3	0.94 (0.71-1.23)	0.637
		TT	39/8.2	42/8.4	0.96 (0.60-1.52)	0.855
*IL-8*		AA	192/40.5	186/37.1	-	-
		AT	231/48.7	213/42.5	1.05 (0.80-1.38)	0.724
		TT	51/10.8	102/20.4	0.48 (0.33-0.72)	<0.001
*IL-10*		CC	186/39.2	234/46.7	-	-
		CT	198/41.8	219/43.7	1.14 (0.87-1.50)	0.354
		TT	90/19.0	48/9.6	2.36 (1.58-3.52)	<0.001
*TNF* c.-418		GG	399/84.2	374/74.7	-	-
		GA	69/14.6	112/22.4	0.58 (0.41-0.80)	0.001
		AA	6/1.3	15/3.0	0.38 (0.14-0.98)	0.044
*TNF* c.-488		GG	404/85.2	397/79.2	-	-
		GA	66/13.9	95/19.0	0.68 (0.48-0.96)	0.029
		AA	4/0.8	9/1.8	0.44 (0.13-1.43)	0.171

### Combinations of polymorphisms and their associations with breast cancer risk

When *NFKB1* and *NFKBIA* polymorphic genotypes were combined, positive ORs were observed for all the combinations. However, 5 out of 8 combinations showed significant association with breast cancer risk (Table 4) and only three combinations of *IL-8* and *IL-10* polymorphisms showed significant association with breast cancer risk (Table 4). Only four combinations of *TNF* c.-418 and c.-488 were analyzed due to the absence of other combinations in the study participants and two of them showed a significant association with breast cancer risk (Table 4).

**Table 4:** Combination of polymorphisms and their associations with breast cancer risk in cases with breast cancer and control participants

Genotype combination		Cases	Controls	Odds ratio (95% confidence interval)	*P*
*NFKB1* ins/ins	*NFKBIA* CC	50	92	-	-
*NFKB1* ins/del	*NFKBIA* CC	127	127	1.84 (1.21-2.81)	0.004
*NFKB1* del/del	*NFKBIA* CC	111	78	2.62 (1.67-4.11)	<0.001
*NFKB1* ins/ins	*NFKBIA* CT	33	57	1.07 (0.61-1.85)	0.822
*NFKB1* ins/del	*NFKBIA* CT	66	68	1.79 (1.10-2.89)	0.019
*NFKB1* del/del	*NFKBIA* CT	46	37	2.29 (1.32-3.98)	0.003
*NFKB1* ins/ins	*NFKBIA* TT	9	13	1.27 (0.51-3.19)	0.604
*NFKB1* ins/del	*NFKBIA* TT	16	21	1.40 (0.67-2.93)	0.369
*NFKB1* del/del	*NFKBIA* TT	14	8	3.22 (1.26-8.20)	0.014
*IL-8* AA	*IL-10* CC	76	93	-	-
*IL-8* AT	*IL-10* CC	78	93	1.03 (0.67-1.57)	0.905
*IL-8* TT	*IL-10* CC	32	48	0.82 (0.48-1.40)	0.460
*IL-8* AA	*IL-10* CT	82	75	1.34 (0.87-2.07)	0.191
*IL-8* AT	*IL-10* CT	101	101	1.22 (0.81-1.84)	0.334
*IL-8* TT	*IL-10* CT	13	43	0.37 (0.19-0.74)	0.005
*IL-8* AA	*IL-10* TT	33	18	2.24 (1.17-4.29)	0.014
*IL-8* AT	*IL-10* TT	48	19	3.09 (1.68-5.70)	<0.001
*IL-8* TT	*IL-10* TT	6	11	0.67 (0.24-1.89)	0.446
*TNF* c.-418 GG	*TNF* c.-488 GG	399	374	-	-
*TNF* c.-418 GA	*TNF* c.-488 GG	3	17	0.17 (0.05-0.57)	0.004
*TNF* c.-418 AA	*TNF* c.-488 GG	2	6	0.31 (0.62-1.56)	0.156
*TNF* c.-418 GG	*TNF* c.-488 GA	0	0	N/A	N/A
*TNF* c.-418 GA	*TNF* c.-488 GA	66	95	0.65 (0.46-0.92)	0.014
*TNF* c.-418 AA	*TNF* c.-488 GA	0	0	N/A	N/A
*TNF* c.-418 GG	*TNF* c.-488 AA	0	0	N/A	N/A
*TNF* c.-418 GA	*TNF* c.-488 AA	0	0	N/A	N/A
*TNF* c.-418 AA	*TNF* c.-488 AA	4	9	0.42 (0.13-1.36)	0.148

### Stratification of breast cancer risk association according to menopausal status

For pre-menopausal women, significant associations with breast cancer risk were observed for *NFKB1* ins/del and del/del genotypes, *NFKBIA* CT genotype, *IL-8* TT genotype, *IL-10* TT genotype, and *TNF* c.-418 GA and AA genotypes. For post-menopausal women, significant associations with breast cancer risk were observed for *NFKB1* ins/del and del/del genotypes, *IL-8* TT genotype, *IL-10* TT genotype, *TNF* c.-418 GA and AA genotypes, and *TNF* c.-488 GA genotype (Table 5).

**Table 5:** Association between the polymorphisms and breast cancer risk among pre- and post-menopausal women with and without breast cancer

Menopause	Genotype	Cases	Controls	Odds ratio (95% confidence interval)	*P*
Pre	*NFKB1* ins/ins	34	67	-	-
Pre	*NFKB1* ins/del	84	90	1.84 (1.11-3.06)	0.019
Pre	*NFKB1* del/del	61	56	2.15 (1.24-3.72)	0.006
Post	*NFKB1* ins/ins	59	95	-	-
Post	*NFKB1* ins/del	126	126	1.61 (1.07-2.42)	0.022
Post	*NFKB1* del/del	110	67	2.64 (1.69-4.12)	<0.001
Pre	*NFKBIA* CC	119	124	-	-
Pre	*NFKBIA* CT	44	73	0.63 (0.40-0.99)	0.043
Pre	*NFKBIA* TT	16	16	1.04 (0.50-2.18)	0.913
Post	*NFKBIA* CC	169	173	-	-
Post	*NFKBIA* CT	103	89	1.18 (0.83-1.69)	0.348
Post	*NFKBIA* TT	23	26	0.91 (0.50-1.65)	0.746
Pre	*IL-8* AA	72	79	-	-
Pre	*IL-8* AT	85	86	1.08 (0.70-1.68)	0.717
Pre	*IL-8* TT	22	48	0.50 (0.28-0.91)	0.024
Post	*IL-8* AA	120	107	-	-
Post	*IL-8* AT	140	127	0.98 (0.69-1.40)	0.924
Post	*IL-8* TT	29	54	0.48 (0.28-0.81)	0.006
Pre	*IL-10* CC	73	104	-	-
Pre	*IL-10* CT	72	92	1.11 (0.73-1.71)	0.620
Pre	*IL-10* TT	28	17	2.35 (1.20-4.60)	0.013
Post	*IL-10* CC	113	130	-	-
Post	*IL-10* CT	120	127	1.09 (0.76-1.55)	0.644
Post	*IL-10* TT	62	31	2.30 (1.40-3.79)	0.011
Pre	*TNF* c.-418 GG	150	162	-	-
Pre	*TNF* c.-418 GA	26	45	0.62 (0.37-1.06)	0.082
Pre	*TNF* c.-418 AA	3	6	0.54 (0.13-2.20)	0.389
Post	*TNF* c.-418 GG	249	212	-	-
Post	*TNF* c.-418 GA	43	67	0.55 (0.36-0.84)	0.005
Post	*TNF* c.-418 AA	3	9	0.28 (0.08-1.06)	0.061
Pre	*TNF* c.-488 GG	154	173	-	-
Pre	*TNF* c.-488 GA	24	37	0.73 (0.42-1.27)	0.266
Pre	*TNF* c.-488 AA	1	3	0.37 (0.04-3.64)	0.397
Post	*TNF* c.-488 GG	252	224	-	-
Post	*TNF* c.-488 GA	41	58	0.63 (0.41-0.97)	0.038
Post	*TNF* c.-488 AA	2	6	0.30 (0.06-1.48)	0.139

### Risk association according to patient histopathological types

*NFKB1* heterozygous and variant genotypes were associated with breast cancer risk in invasive ductal carcinoma (IDC) and ductal carcinoma in situ (DCIS), but not in invasive lobular carcinoma (ILC). *IL10* variant genotype was associated with increased breast cancer risk in all three types of breast cancers. On the other hand, *IL8* variant genotype and heterozygous genotypes of both *TNF* polymorphisms were associated with decreased risk of IDC but not of other types of breast cancer (Table 6).

**Table 6:** Association between the polymorphisms and breast cancer risk according to histopathological type of patients

Histo-pathological type*	Genotype	Cases	Controls	Odds ratio (95% confidence interval)	*P*
IDC	*NFKB1* ins/ins	64	162	-	-
IDC	*NFKB1* ins/del	152	216	1.78 (1.25-2.54)	0.002
IDC	*NFKB1* del/del	130	123	2.68 (1.83-3.91)	<0.001
DCIS	*NFKB1* ins/ins	12	162	-	-
DCIS	*NFKB1* ins/del	33	216	2.06 (1.03-4.12)	0.040
DCIS	*NFKB1* del/del	26	123	2.85 (1.38-5.88)	0.005
ILC	*NFKB1* ins/ins	17	162	-	-
ILC	*NFKB1* ins/del	25	216	1.10 (0.58-2.11)	0.767
ILC	*NFKB1* del/del	15	123	1.16 (0.56-2.42)	0.688
IDC	*NFKBIA* CC	212	297	-	-
IDC	*NFKBIA* CT	102	162	0.88 (0.65-1.19)	0.419
IDC	*NFKBIA* TT	32	42	1.07 (0.65-1.75)	0.795
DCIS	*NFKBIA* CC	46	297	-	-
DCIS	*NFKBIA* CT	25	162	0.99 (0.59-1.68)	0.989
DCIS	*NFKBIA* TT	0	42	N/A	N/A
ILC	*NFKBIA* CC	30	297	-	-
ILC	*NFKBIA* CT	20	162	1.22 (0.67-2.22)	0.510
ILC	*NFKBIA* TT	7	42	1.65 (0.68-3.99)	0.266
	*IL-8* AA	137	186	-	-
IDC	*IL-8* AT	174	213	1.10 (0.82-1.49)	0.496
IDC	*IL-8* TT	35	102	0.46 (0.29-0.72)	0.001
DCIS	*IL-8* AA	29	186	-	-
DCIS	*IL-8* AT	33	213	0.99 (0.58-1.69)	0.981
DCIS	*IL-8* TT	9	102	0.56 (0.25-1.24)	0.156
ILC	*IL-8* AA	26	186	-	-
ILC	*IL-8* AT	24	213	0.80 (0.44-1.45)	0.473
ILC	*IL-8* TT	7	102	0.49 (0.20-1.17)	0.108
IDC	*IL-10* CC	140	234	-	-
IDC	*IL-10* CT	147	219	1.12 (0.83-1.50)	0.446
IDC	*IL-10* TT	59	48	2.05 (1.33-3.17)	0.001
DCIS	*IL-10* CC	29	234	-	-
DCIS	*IL-10* CT	29	219	1.06 (0.61-1.84)	0.812
DCIS	*IL-10* TT	13	48	2.18 (1.05-4.50)	0.034
ILC	*IL-10* CC	17	234	-	-
ILC	*IL-10* CT	22	219	1.38 (0.71-2.67)	0.335
ILC	*IL-10* TT	18	48	5.16 (2.48-10.73)	<0.001
IDC	*TNF* c.-418 GG	298	374	-	-
IDC	*TNF* c.-418 GA	43	112	0.48 (0.32-0.71)	0.001
IDC	*TNF* c.-418 AA	5	15	0.41 (0.15-1.16)	0.095
DCIS	*TNF* c.-418 GG	61	374	-	-
DCIS	*TNF* c.-418 GA	10	112	0.54 (0.27-1.10)	0.092
DCIS	*TNF* c.-418 AA	0	15	N/A	N/A
ILC	*TNF* c.-418 GG	40	374	-	-
ILC	*TNF* c.-418 GA	16	112	1.33 (0.72-2.47)	0.358
ILC	*TNF* c.-418 AA	1	15	0.62 (0.08-4.84)	0.651
IDC	*TNF* c.-488 GG	302	397	-	-
IDC	*TNF* c.-488 GA	41	95	0.56 (0.38-0.84)	0.005
IDC	*TNF* c.-488 AA	3	9	0.43 (0.11-1.63)	0.219
DCIS	*TNF* c.-488 GG	61	397	-	-
DCIS	*TNF* c.-488 GA	10	95	0.68 (0.33-1.386)	0.293
DCIS	*TNF* c.-488 AA	0	9	N/A	N/A
ILC	*TNF* c.-488 GG	41	397	-	-
ILC	*TNF* c.-488 GA	15	95	1.52 (0.81-2.87)	0.188
ILC	*TNF* c.-488 AA	1	9	1.07 (0.13-8.70)	0.945

*IDC – invasive ductal carcinoma; DCIS – ductal carcinoma in situ; ILC – invasive lobular carcinoma.

### Risk association according to patient cancer grading

Increased risk associations were observed for *NFKB1* heterozygous genotype (in Grade 2 and 3 patients), *NFKB1* variant genotype (in all patients), *NFKBIA* variant genotype (in Grade 1 patients), *IL10* heterozygous genotype (in Grade 1 patients), *IL10* variant genotype (in all patients), and *TNF* c.488 heterozygous genotype (in Grade 1 patients). Decreased risk associations were observed for *IL8* heterozygous and variant genotypes, *TNF* c.418 heterozygous genotype, and *TNF* c.488 heterozygous genotype (all in Grade 2 and 3 patients) (Table 7).

**Table 7:** Association between the polymorphisms and breast cancer risk according to cancer grading of patients

Grade*	Genotype	Cases	Controls	Odds ratio (95% confidence interval)	*P*
1	*NFKB1* ins/ins	10	162	-	-
1	*NFKB1* ins/del	12	216	0.90 (0.37-2.13)	0.811
1	*NFKB1* del/del	20	123	2.63 (1.19-5.83)	0.017
2	*NFKB1* ins/ins	44	162	-	-
2	*NFKB1* ins/del	101	216	1.72 (1.14-2.59)	0.009
2	*NFKB1* del/del	83	123	2.48 (1.60-3.83)	<0.001
3	*NFKB1* ins/ins	39	162	-	-
3	*NFKB1* ins/del	97	216	1.86 (1.22-2.84)	0.004
3	*NFKB1* del/del	68	123	2.29 (1.45-3.63)	<0.001
1	*NFKBIA* CC	14	297	-	-
1	*NFKBIA* CT	16	162	2.09 (0.99-4.40)	0.051
1	*NFKBIA* TT	12	42	6.06 (2.62-13.98)	<0.001
2	*NFKBIA* CC	144	297	-	-
2	*NFKBIA* CT	67	162	0.81 (0.57-1.15)	0.253
2	*NFKBIA* TT	17	42	0.83 (0.45-1.51)	0.554
3	*NFKBIA* CC	130	297	-	-
3	*NFKBIA* CT	64	162	0.90 (0.63-1.28)	0.571
3	*NFKBIA* TT	10	42	0.54 (0.26-1.11)	0.097
1	*IL-8* AA	17	186	-	-
1	*IL-8* AT	16	213	0.82 (0.40-1.67)	0.588
1	*IL-8* TT	9	102	0.96 (0.41-2.24)	0.935
2	*IL-8* AA	90	186	-	-
2	*IL-8* AT	111	213	0.54 (0.33-0.89)	0.017
2	*IL-8* TT	27	102	1.07 (0.76-1.51)	0.669
3	*IL-8* AA	85	186	-	-
3	*IL-8* AT	104	213	1.06 (0.75-1.51)	0.709
3	*IL-8* TT	15	102	0.32 (0.17-0.58)	<0.001
1	*IL-10* CC	5	234	-	-
1	*IL-10* CT	28	219	5.98 (2.26-15.77)	<0.001
1	*IL-10* TT	9	48	8.77 (2.81-27.34)	<0.001
2	*IL-10* CC	91	234	-	-
2	*IL-10* CT	91	219	1.06 (0.75-1.50)	0.706
2	*IL-10* TT	46	48	2.46 (1.53-3.94)	<0.001
3	*IL-10* CC	90	234	-	-
3	*IL-10* CT	79	219	0.93 (0.65-1.33)	0.722
3	*IL-10* TT	35	48	1.89 (1.15-3.12)	0.012
1	*TNF* c.-418 GG	30	374	-	-
1	*TNF* c.-418 GA	12	112	1.33 (0.66-2.69)	0.419
1	*TNF* c.-418 AA	0	15	N/A	N/A
2	*TNF* c.-418 GG	190	374	-	-
2	*TNF* c.-418 GA	33	112	0.58 (0.37-0.88)	0.012
2	*TNF* c.-418 AA	5	15	0.65 (0.23-1.83)	0.421
3	*TNF* c.-418 GG	179	374	-	-
3	*TNF* c.-418 GA	24	112	0.44 (0.27-0.72)	0.001
3	*TNF* c.-418 AA	1	15	0.13 (0.01-1.06)	0.057
1	*TNF* c.-488 GG	21	397	-	-
1	*TNF* c.-488 GA	21	95	4.17 (2.19-7.96)	<0.001
1	*TNF* c.-488 AA	0	9	N/A	N/A
2	*TNF* c.-488 GG	199	397	-	-
2	*TNF* c.-488 GA	27	95	0.56 (0.35-0.89)	0.016
2	*TNF* c.-488 AA	2	9	0.44 (0.09-2.07)	0.301
3	*TNF* c.-488 GG	184	397	-	-
3	*TNF* c.-488 GA	18	95	0.40 (0.23-0.69)	0.001
3	*TNF* c.-488 AA	2	9	0.48 (0.10-2.24)	0.350

*Grade 1 – well differentiated; Grade 2 – moderately differentiated; Grade 3 – poorly differentiated.

## Discussion

This study established that the ins/del and del/del genotypes of NFKB1 polymorphism and TT genotype of IL-10 polymorphism significantly increased breast cancer risk, while the TT genotype of IL-8 polymorphism, GA and AA genotypes of TNF c.-418G>A polymorphism, and GA genotype of TNF c.-488G>A polymorphism significantly reduced breast cancer risk. Various lines of evidence have found that chronic inflammation was a risk factor for breast cancer development (16-18). Inflammation can cause DNA damage, and hence carcinogenesis, by inducing and activating oxidant-producing enzymes (19). Events that are linked to inflammation, such as postmenopausal status and obesity, have also been associated with an increased breast cancer risk (6). If inflammation represents an important pathway in carcinogenesis, polymorphisms in the inflammatory response genes could potentially modify cancer predisposition risk.

We analyzed not only the association of individual polymorphisms and breast cancer risk, but also the effects of combinations of functionally related polymorphisms (*NFKB1* and *NFKBIA*; *IL-8* and *IL-10*; and *TNF* c.-418 and c.-488), menopausal status, histopathological type, and cancer grading. To our knowledge, this is the first study investigating the association between *NFKB1* polymorphism and breast cancer risk although there are a few reports on its association with several other cancers. Our findings are in agreement with a study from East China that found that del/del genotype increased the risk of bladder cancer (20). However, a study in Southern Chinese population (21) found that the ins/ins genotype increased the risk of colorectal cancer. Our report also presents the first evidence for the association of *NFKBIA* polymorphism with the risk of breast cancer in any Asian population. Thus far, only one study has examined this association but it was conducted in a Caucasian population (22). Similarly to our study, they found no association between *NFKBIA* polymorphism and breast cancer risk. For *IL-8* polymorphism, one study conducted in East China showed no association with breast cancer risk (23). Our results are in disagreement with this study, whose genotype distribution deviate significantly from the Hardy-Weinberg equilibrium. However, our results are similar to an Iranian study, which also found an association between the variant genotype of the polymorphism and breast cancer risk (24). On the other hand, a study from East China showed no association between *IL-10* polymorphism and breast cancer risk (25), which is different from our results. For *TNF* c.-418 and c.-488 polymorphisms, an Indian study (26), reported that the AA genotype resulted in an increased breast cancer risk, which is also different from our results. It should be noted, however, that this study had a small sample size with only 40 cases. Similar to our study, Park et al (27) reported a reduced risk of breast cancer among carriers of the A allele of the polymorphisms. However, this risk reduction was not statistically significant.

In conclusion, our study provided evidence for the association of various inflammatory response gene polymorphisms with the risk of breast cancer in East China. The strengths of the present study are the reasonably large sample size and the detailed combination and stratification analyses performed. The limitations of the study are the small number of polymorphisms studied within each gene and the small sample sizes obtained by stratification according to menopausal status, histopathological type, and cancer grading, which might have led to misleading interpretation. Therefore, further studies by independent research groups are needed to confirm our findings.

## References

[1] Jemal A, Bray F, Center MM, Ferlay J, Ward E, Forman D (2011). Global cancer statistics.. CA Cancer J Clin.

[2] Ferlay J, Shin HR, Bray F, Forman D, Mathers C, Parkin DM. GLOBOCAN 2008 v2.0, Cancer Incidence and Mortality Worldwide: IARC CancerBase No. 10 (Internet). Lyon: International Agency for Research on Cancer; 2010.

[3] Porter P (2008). “Westernizing” women’s risks? Breast cancer in lower-income countries.. N Engl J Med.

[4] Njiaju UO, Olopade OI (2012). Genetic determinants of breast cancer risk: a review of current literature and issues pertaining to clinical application.. Breast J.

[5] Newman B, Austin MA, Lee M, King M (1988). Inheritance of human breast cancer: evidence for autosomal dominant transmission in high-risk families.. Proc Natl Acad Sci U S A.

[6] Mohd Suzairi MS, Tan SC, Ahmad Aizat AA, Mohd Aminudin M, Siti Nurfatimah MS, Andee ZD (2013). The functional -94 insertion/deletion ATTG polymorphism in the promoter region of NFKB1 gene increases the risk of sporadic colorectal cancer.. Cancer Epidemiol..

[7] Tan SC, Suzairi MS, Aizat AA, Aminudin MM, Nurfatimah MS, Bhavaraju VM (2013). Gender-specific association of NFKBIA promoter polymorphisms with the risk of sporadic colorectal cancer.. Med Oncol.

[8] Song B, Zhang D, Wang S, Zheng H, Wang X (2009). Association of interleukin-8 with cachexia from patients with low-third gastric cancer.. Comp Funct Genomics.

[9] Lajin B, Hamzeh AR, Ghabreau L, Mohamed A, Moustafa AA, Alachkar A (2013). Catechol-O-methyltransferase Val 108/158 Met polymorphism and breast cancer risk: a case control study in Syria.. Breast Cancer.

[10] Wang J, Guo X, Zhang J, Song J, Ji M, Yu S (2013). Cyclooxygenase-2 polymorphisms and susceptibility to colorectal cancer: a meta-analysis.. Yonsei Med J.

[11] Pooja S, Francis A, Bid HK, Kumar S, Rajender S, Ramalingam K (2011). Role of ethnic variations in TNF-α and TNF-β polymorphisms and risk of breast cancer in India.. Breast Cancer Res Treat.

[12] Constantinou C, Fentiman IS (2013). Inflammation and breast cancer.. Breast Cancer Management..

[13] Abraham LJ, Kroeger KM (1999). Impact of the -308 TNF promoter polymorphism on the transcriptional regulation of the TNF gene: relevance to disease.. J Leukoc Biol.

[14] Mohebbatikaljahi H, Menevse S, Yetkin I, Demirci H (2009). Study of interleukin-10 promoter region polymorphisms (-1082A/G, -819T/C and -592A/C) in type 1 diabetes mellitus in Turkish population.. J Genet.

[15] Stayoussef M, Benmansour J, Al-Jenaidi FA, Rajab MH, Said HB, Ourtani M (2010). Identification of specific tumor necrosis factor-α-susceptible and -protective haplotypes associated with the risk of type 1 diabetes.. Eur Cytokine Netw.

[16] DeNardo DG, Coussens LM (2007). Inflammation and breast cancer. Balancing immune response: crosstalk between adaptive and innate immune cells during breast cancer progression.. Breast Cancer Res.

[17] Baumgarten SC, Frasor J (2012). Minireview: Inflammation: an instigator of more aggressive estrogen receptor (ER) positive breast cancers.. Mol Endocrinol.

[18] Macciň A, Madeddu C (2011). Obesity, inflammation, and postmenopausal breast cancer: therapeutic implications.. ScientificWorldJournal.

[19] Murata M, Thanan R, Ma N, Kawanishi S (2012). Role of nitrative and oxidative DNA damage in inflammation-related carcinogenesis.. J Biomed Biotechnol.

[20] Li P, Gu J, Yang X, Cai H, Tao J, Yang X (2013). Functional promoter -94 ins/del ATTG polymorphism in NFKB1 gene is associated with bladder cancer risk in a Chinese population.. PLoS ONE.

[21] Song S, Chen D, Lu J, Liao J, Luo Y, Yang Z (2011). NFκB1 and NFκBIA polymorphisms are associated with increased risk for sporadic colorectal cancer in a southern Chinese population.. PLoS ONE.

[22] Curran JE, Weinstein SR, Griffiths LR (2002). Polymorphic variants of NFKB1 and its inhibitory protein NFKBIA, and their involvement in sporadic breast cancer.. Cancer Lett.

[23] Liu JY, Zhai XJ, Jin GF (2007). A study of relationship between polymorphisms of interleukin-8 and risk of breast cancer in Chinese population.. Bulletin of Chinese Cancer..

[24] Kamali-Sarvestani E, Aliparasti MR, Atefi S (2007). Association of interleukin-8 (IL-8 or CXCL8) -251T/A and CXCR2 +1208C/T gene polymorphisms with breast cancer.. Neoplasma.

[25] Kong F, Liu J, Liu Y, Song B, Wang H, Liu W (2010). Association of interleukin-10 gene polymorphisms with breast cancer in a Chinese population.. J Exp Clin Cancer Res.

[26] Kohaar I, Tiwari P, Kumar R, Nasare V, Thakur N, Das BC (2009). Association of single nucleotide polymorphisms (SNPs) in TNF-LTA locus with breast cancer risk in Indian population.. Breast Cancer Res Treat.

[27] Park KS, Mok JW, Ko HE, Tokunaga K, Lee MH (2002). Polymorphisms of tumour necrosis factors A and B in breast cancer.. Eur J Immunogenet.

